# Non-invasive prediction of the mouse tibia mechanical properties from microCT images: comparison between different finite element models

**DOI:** 10.1007/s10237-021-01422-y

**Published:** 2021-02-01

**Authors:** S. Oliviero, M. Roberts, R. Owen, G. C. Reilly, I. Bellantuono, E. Dall’Ara

**Affiliations:** 1 Department of Oncology and Metabolism, Mellanby Centre for Bone Research, University of Sheffield, Sheffield, UK; 2 INSIGNEO Institute for in Silico Medicine, University of Sheffield, Sheffield, UK; 3 Department of Mechanical Engineering, University of Sheffield, Sheffield, UK; 4 Department of Materials Science and Engineering, University of Sheffield, Sheffield, UK; 5 Regenerative Medicine and Cellular Therapies, School of Pharmacy, University of Nottingham Biodiscovery Institute, University Park, UK; 6 Healthy Lifespan Institute, The Medical School, University of Sheffield, Sheffield, UK

**Keywords:** Mouse tibia, MicroCT, Validation, Stiffness, Failure load, Finite element

## Abstract

New treatments for bone diseases require testing in animal models before clinical translation, and the mouse tibia is among the most common models. In vivo micro-Computed Tomography (microCT)-based micro-Finite Element (microFE) models can be used for predicting the bone strength non-invasively, after proper validation against experimental data. Different modelling techniques can be used to estimate the bone properties, and the accuracy associated with each is unclear. The aim of this study was to evaluate the ability of different microCT-based microFE models to predict the mechanical properties of the mouse tibia under compressive load. Twenty tibiae were microCT scanned at 10.4 µm voxel size and subsequently compressed at 0.03 mm/s until failure. Stiffness and failure load were measured from the load–displacement curves. Different microFE models were generated from each microCT image, with hexahedral or tetrahedral mesh, and homogeneous or heterogeneous material properties. Prediction accuracy was comparable among models. The best correlations between experimental and predicted mechanical properties, as well as lower errors, were obtained for hexahedral models with homogeneous material properties. Experimental stiffness and predicted stiffness were reasonably well correlated (*R*^2^ = 0.53–0.65, average error of 13–17%). A lower correlation was found for failure load (*R*^2^ = 0.21–0.48, average error of 9–15%). Experimental and predicted mechanical properties normalized by the total bone mass were strongly correlated (*R*^2^ = 0.75–0.80 for stiffness, *R*^2^ = 0.55–0.81 for failure load). In conclusion, hexahedral models with homogeneous material properties based on in vivo microCT images were shown to best predict the mechanical properties of the mouse tibia.

**Supplementary Information:**

The online version contains supplementary material available at. 10.1007/s10237-021-01422-y.

## Introduction

Osteoporosis and osteoarthritis are the most common chronic diseases of the musculoskeletal system, and therefore the development of new bone physical and/or pharmacological treatments is needed. Before clinical translation, testing on animal models is required, and the mouse tibia is among the most commonly observed anatomical sites for evaluating bone properties (Bouxsein et al. 2010). The main advantages of mouse models are the possibility to control the animal environment, to perform high-resolution assessment of bone and other musculoskeletal tissues, and the relatively low costs. In particular, micro-Computed Tomography (microCT) imaging is extensively used to measure the bone microstructure and density, as well as their changes over time (Bouxsein et al. 2010). Osteoporotic patients show lower bone mineral density in central sites, with reduced mechanical properties and increased risk of fracture (Viceconti and Dall’Ara 2019). Therefore, for improving clinical translation, the bone mechanical properties should be measured in mouse disease models before and after treatments. However, the experimental measurement of bone strength is invasive and cannot be performed in vivo; therefore, other tools have been developed for its estimation. Finite Element (FE) models based on CT or microCT images have been applied and validated for the prediction of structural mechanical properties of different bone types (Zysset et al. 2013), such as trabecular bone specimens (Schwiedrzik et al. 2016; Wolfram et al. 2010), human vertebral bodies (Crawford et al. 2003; Dall’Ara et al. 2012; Gustafson et al. 2017), human femur (Dall'Ara et al. 2013; Pottecher et al. 2016; Schileo et al. 2008) and human distal radius (Macneil and Boyd 2008; Pistoia et al. 2002; Varga et al. 2011). Similarly, in preclinical studies, FE models have been used for predicting the structural mechanical properties of the mouse vertebra and femur (Nyman et al. 2015; Varga et al. 2020) or the strain distributions in the mouse tibia under loading (Birkhold et al. 2016; Yang et al. 2017). This approach, combined with a longitudinal experimental design (Dall’Ara et al. 2016), has the potential to dramatically reduce the usage of mice in bone research, a fundamental step towards the 3Rs (replacement, refinement and reduction of the usage of animals in research) (Viceconti and Dall’Ara 2019). Nevertheless, the validation of FE models of mouse bones against experimental data is limited and it is unclear whether accounting for heterogeneous material properties (differences in local tissue mineral density within the bone) (Gross et al. 2012) and recovering the realistic boundary of the structure with smooth models would improve the predictive ability of the models (Table 1). Nyman et al. (2015) reported that hexahedral microFE models with homogeneous or heterogeneous material properties could predict the mouse vertebra strength in compression, even though the accuracy was dependant on the assigned material properties and on the chosen failure parameters (*R*^2^ = 0.62–0.89, Table 1). Varga et al. (2020) found a strong correlation between the mouse femur failure load measured in four-point bending and predictions by linear homogeneous microFE models with hexahedral mesh after optimization of the failure criterion (*R*^2^ = 0.93) (Varga et al. 2020). In the above studies, failure load was obtained from linear microFE models by assuming that the bone fails when a portion of the nodes (failure volume) reaches a critical strain level (adapted from Pistoia et al. 2002). The optimal failure volume and critical strain level were optimized against experimental data. For the mouse tibia, a few studies have reported a comparison between the predictions of local displacements and strains from FE models and experimental measurements. Local strains measured with strain gauges have been compared to those predicted by homogeneous or heterogeneous microFE models with hexahedral or tetrahedral mesh at the corresponding spatial locations (Patel et al. 2014; Razi et al. 2015; Stadelmann et al. 2009; Yang et al. 2014), showing differences in the range of 1–48% (Table 1). Sensitivity analyses were carried out to evaluate the influence of different modelling parameters on the strain predictions, including scan resolution, mesh refinement, material properties and boundary conditions (Razi et al. 2014; Yang et al. 2014). However, strain gauge measurements can only be acquired in a limited number of spatial locations over the tibia, and the application of the sensor may cause a local stiffening of the specimen, as shown for the mouse forearm (Begonia et al. 2017). Digital Image Correlation (DIC) measurements have also been used to qualitatively validate microFE strain distributions on the surface of the tibia (Pereira et al. 2015). In Pereira et al. (2015), the local mechanical stimulus (interstitial fluid velocity) obtained from poroelastic models was used to simulate the bone adaptation driven by compressive loading. However, in the above studies, structural mechanical properties were not estimated from the models or compared to experimental measurements. A previous study performed by our group showed that hexahedral homogeneous microFE models based on in vivo microCT images can predict well (*R*^2^ > 0.82) the local displacements across the tibia volume measured with Digital Volume Correlation (Oliviero et al. 2018). The microFE models were found to accurately predict the apparent stiffness (errors of 14% ± 11%) and failure load (errors of 9% ± 9%). However, sample size was limited (*N* = 6) due to the complex validation method (microCT imaging combined with in situ mechanical testing) and mechanical properties were estimated from stepwise mechanical tests with limited control of the loading rate. To the authors’ knowledge, only a previous study by our group has reported the ability of linear homogeneous microFE models with hexahedral mesh in predicting the structural properties of the mouse tibia (stiffness and failure load) (Oliviero et al. 2021). In that study, after the optimization of the failure criterion for the mouse tibia, based on a previous approach used for the human distal radius (Pistoia et al. 2002), microFE models were found to predict the structural mechanical properties fairly well (apparent stiffness: *R*^2^ = 0.65, errors of 14% ± 8%; failure load: *R*^2^ = 0.48; errors of 9% ± 6%) and the normalized mechanical properties very well (normalized stiffness: R^2^ = 0.80, errors of 14% ± 8%; normalized failure load: *R*^2^ = 0.81, errors of 9% ± 6%). Nevertheless, the predictive ability of more complex models that would account for heterogeneous bone properties or that would recover the smooth boundary of the bone was not tested against experimental measurements of structural properties. Therefore, it is still unknown which modelling approach would better predict the structural mechanical properties of the mouse tibia.

**Table 1:** Summary of the validation studies reported in the literature for mouse bones

Reference	Site	Sample size	Voxel size (μm)	Loading condition	Exp meas	Mesh type	Mesh size (μm) or number of elements	Material properties	Failure criterion	Validation parameter	Regression parameters	Error (%)
Stadelmann et al. (2009)	Tibia	18	9	Compression	Strain gauge	Hexa	26,000 elements	Hete *E* = − 3.842 + 0.013TMD	N/A	Strain	N/A	< 10%
Patel et al. (2014)	Tibia	12	21	Compression	Strain gauge	Hexa	6 M elements	Homo *E* = 21GPa	N/A	Strain	N/A	8–48%
Yang et al. (2014)	Tibia	6	10	Compression	In vivo Strain gauge	Tetra	1.5 M elements	Hete E = 1.127* 10^−4^TMD^1.746^	N/A	Strain	R^2^ = 0.66 Slope = 1.01 Int = -112µε	N/A
Razi et al. (2015)	Tibia	15	9.9	Compression	In vivo Strain gauge	Tetra	52 μm (average)	Homo *E* = 17GPa	N/A	Strain	N/A	< 20%
						Tetra	52 μm (average)	Hete *E*/*E*_max_ = (µ/µ_max_)^1.5^ E_max_ = 17GPa	N/A	Strain	N/A	< 5%
Pereira et al. (2015)	Tibia	6	5	Compression	DIC	Tetra	47.39 μm (average)	Homo, elastic (*E* = 17GPa) and poroelastic	N/A	Strain	Qualitative good agreement of strain distributions	
Begonia et al. (2017)	Forearm	3	10.5	Compression	Strain gauge	Tetra	140,000–200,000 elements	Homo, specimen-specific Ulna: *E* = 19–22GPa Radius: *E* = 12–15GPa	N/A	Strain	N/A	37–56%
					DIC				N/A	Strain	N/A	Ulna 3–14% Radius 31–38%
Oliviero et al. (2018)	Tibia	6	10.4	Compression	DVC	Hexa	10.4 μm	Homo *E* = 14.8 GPa	N/A	Displacement	*R*^2^ > 0.82 Slope = 0.69–0.95 RMSE = 5–22%	< 36%
Nyman et al. (2015)	Vertebra	15	12	Compression	Fu	Hexa	12 μm	Homo *E* = 18 GPa	*V* = 2–6% *ε*_*y*_ = 0.007–0.009	Fu	*R*^2^ = 0.62–0.70 Slope = 1.37–1.38 RMSE = 5–8 N	N/A
								*E* = 10^A^ *A* = − 8.58 + 4.05*log(B) *B* = 400/(1 + 0.504/TMD)	*V* = 2–7% *ε*_*y*_ = 0.007–0.009	Fu	*R*^2^ = 0.67–0.74 Slope = 1.09–1.38 RMSE = 4–16 N	N/A
								Hete *E* = 1.127* 10^−4^TMD^1.746^	*V* = 2–5% *ε*_*y*_ = 0.007–0.01	Fu	*R*^2^ = 0.70–0.71 RMSE = 4–15 N	N/A
								*E* = 3.883* 10^–9^* TMD^4.05^	*V* = 2% *ε*_*y*_ = 0.007–0.07	Fu	*R*^2^ = 0.67–0.71 RMSE = 5–31 N	N/A
Varga et al. (2020)	Femur	67	9	4-point bending	Fu	Hexa	9 μm	Homo *E* = 15GPa	*V* = 3% *ε*_*y*_ = 0.01	Fu	*R*^2^ = 0.93 Slope = 1.02 Int = 6 N SEE = 8%	N/A
Oliviero et al. (2021)	Tibia	20	10.4	Compression	S	Hexa	10.4 μm	Homo *E* = 14.8GPa	N/A	S	*R*^2^ = 0.65 Slope = 1.02 Int = 9 N/mm	14% ± 8%
					S				N/A	S_norm	*R*^2^ = 0.80 Slope = 1.20 Int = − 3 N/mm/mg	14% ± 8%
					Fu				*V* = 10% *ε*_*y*_ = 0.01442	Fu	*R*^2^ = 0.48 Slope = 0.64 Int = 16 N	9% ± 6%
					Fu				*V* = 10% *ε*_*y*_ = 0.01442	Fu_norm	*R*^2^ = 0.81 Slope = 1.07 Int = 0 N/mg	9% ± 6%

*Exp meas* experimental measurement used for validating the models, *S*  stiffness (N/mm), *S_norm*  stiffness normalized by total bone mineral content (BMC) (N/mm/mg), *Fu*  failure load (N), *Fu_norm* failure load normalized by total BMC (N/mg), *DIC* digital image correlation, *DVC* digital volume correlation, *Hexa *hexahedral mesh, *Tetra* tetrahedral mesh, *homo* homogeneous material properties, *hete *heterogeneous material properties based on tissue mineral density (TMD), *µ* linear attenuation coefficient, *V* failure volume, *ε*_*y*_ yield strain, *RMSE* root-mean-square error, *SEE* standard error of estimate

The aim of this study was to evaluate the ability of different microFE models based on in vivo microCT images in predicting the experimentally measured structural mechanical properties of the mouse tibia under compression. Six different models were compared, characterized by hexahedral or tetrahedral mesh, homogeneous or heterogeneous material properties.

## Materials and methods

**Fig. 1:**
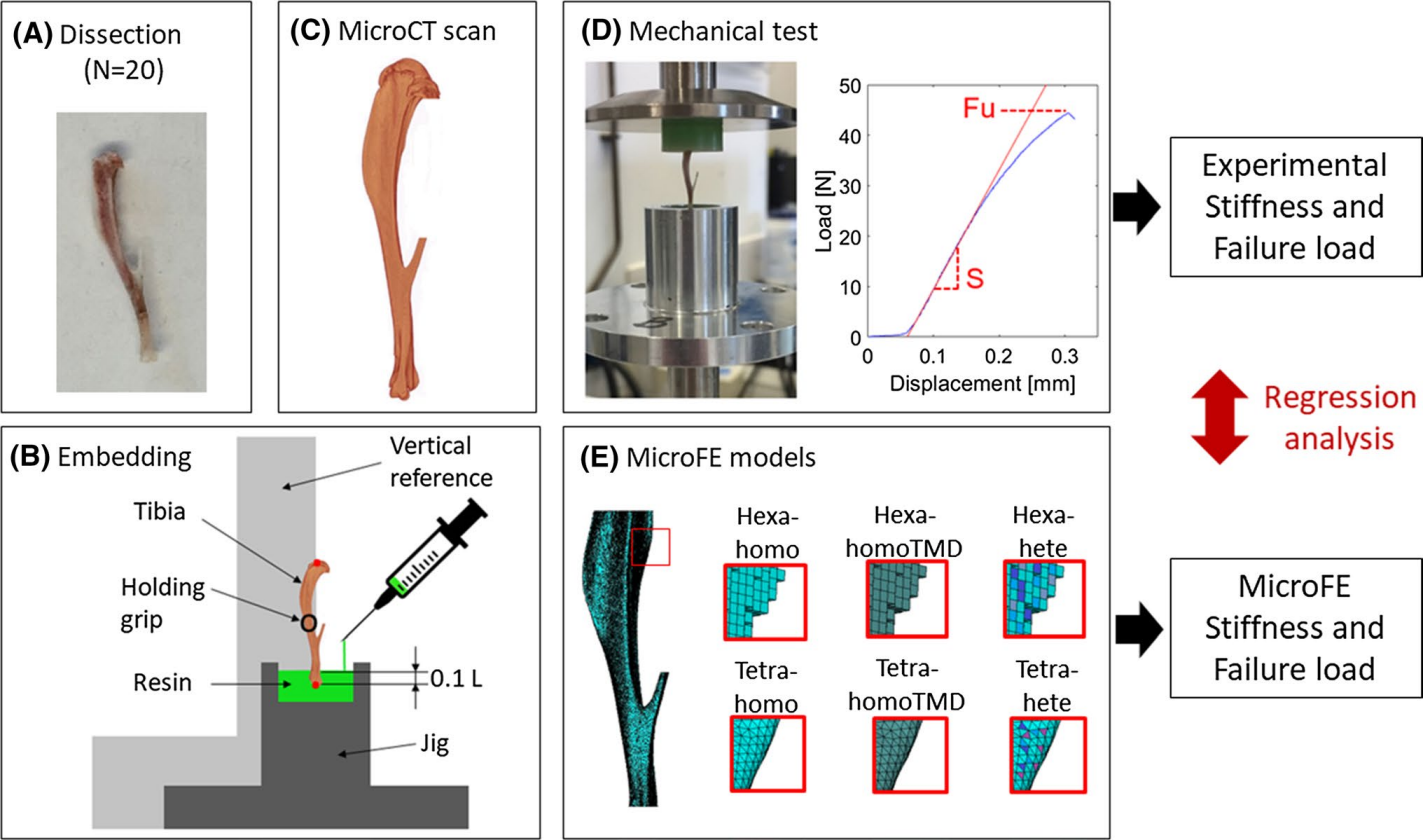
Overview of the methods. Each tibia was dissected (**A**) and the extremities were embedded in resin (**B**). A microCT scan was acquired for each tibia (**C**). The microCT scans were used to generate specimen-specific microFE models (**E**). Subsequently, each tibia was tested in compression (**D**). From the experimental curves, stiffness (S) and failure load (Fu) were measured. Mechanical properties were estimated from the models and compared to the experimental measurements. Regression analysis was used to evaluate the ability of each model to predict the experimental mechanical properties

An overview of the methods used in this study is presented in Fig. 1. In summary, 20 mouse tibiae were microCT scanned and subsequently tested in compression along the longitudinal direction. Apparent stiffness and failure load were measured from the experimental tests. The microCT images were used to generate specimen-specific linear microFE models. Six different models were generated for each tibia: hexahedral mesh with homogeneous material properties (Hexa-homo), hexahedral mesh with specimen-specific homogeneous material properties based on the average Tissue Mineral Density (Hexa-homoTMD), tetrahedral mesh with homogeneous material properties (Tetra-homo), tetrahedral mesh with specimen-specific homogeneous material properties (Tetra-homoTMD), hexahedral mesh with heterogeneous material properties based on the TMD distribution (Hexa-hete) and tetrahedral mesh with heterogeneous material properties (Tetra-hete). From each model, the structural mechanical properties and the normalized structural mechanical properties of each tibia were predicted and compared to the experimental measurements. Details of the experimental measurements and the Hexa-homo microFE models have been reported in Oliviero et al. (2021). The approaches are briefly described below.

### Experimental measurements

Twenty mouse tibiae were collected from female mice of two different strains (C57BL/6J and BALB/c), ages (16 and 24 weeks) and three different intervention groups, used in previous studies (Roberts et al. 2019). Details of the properties of each specimen have been reported previously (Oliviero et al. 2021) and can be found in Supplementary material 1. Intervention groups included in the study were wild-type mice (WT), ovariectomized mice (OVX, surgery performed at week 14 of age) and mice treated with parathyroid hormone (PTH, daily injections, 5 days/week starting from week 18 of age). Both left and right tibiae were included. Specimens from different groups of mice were included in order to increase the range of mechanical properties, thus testing the models in different conditions.

The tibia was isolated from the rest of the leg and its length was measured with a caliper. The longitudinal axis of the tibia was aligned to a vertical reference, and each extremity was embedded in resin (Technovit 4071, Kulzer, Germany) for 10% of the total length (Fig. 1b). The blocks of embedding material were created with a custom-made jig, which served both as a mould for the resin during polymerization and as a grip to mount the specimen on the loading machine. The tibiae were kept frozen at − 20 °C until testing.

Before testing specimens were defrosted and rehydrated in saline solution for 3 h. The bones were wrapped in cling film in order to prevent dehydration during the microCT scan. Each tibia was microCT scanned with a scanning procedure previously defined for in vivo applications (VivaCT 80, Scanco Medical, Bruettisellen, Switzerland; 55 kVp, 145 μA, 10.4 μm voxel size, 100 ms integration time, 32 mm field of view, 750 projections/180°, no frame averaging, 0.5 mm Al filter) (Oliviero et al. 2017, 2019). All images were reconstructed using the software provided by the manufacturer (Scanco Medical AG) and applying a beam hardening correction based on a phantom of 1200 mg HA/cc density, which has been shown to improve the local tissue mineralization measurement (Kazakia et al. 2008).

The bone voxels in each microCT image were identified by using a single-level threshold, calculated as the average of the grey levels corresponding to the bone and background peaks in the histogram of the image (Christiansen 2016; Oliviero et al. 2017). The attenuation coefficients in the bone voxels were converted into tissue mineral density (TMD) by using the calibration law provided by the manufacturer of the scanner. Weekly quality checks were carried out using a densitometric phantom with five insertions (800, 400, 200, 100 and 0 mg HA/cc) in order to monitor the stability of the calibration parameters. The values of equivalent TMD within each insertion computed with the calibration law were compared with the known values for each insertion. According to the manufacturer’s guidelines, the calibration law was not changed if values for the denser insertion were within ± 2% from the nominal value. In case the value was out of range, the operating parameters of the X-rays source were adjusted by the manufacturer and a new calibration law was used. Bone mineral content (BMC) in each voxel was calculated as its TMD multiplied by the volume of the voxel. A volume of interest (VOI) was selected by excluding the portions embedded in the resin (Fig. 1e). Total BMC was computed as the sum of BMC in each bone voxel.

**Fig. 2:**
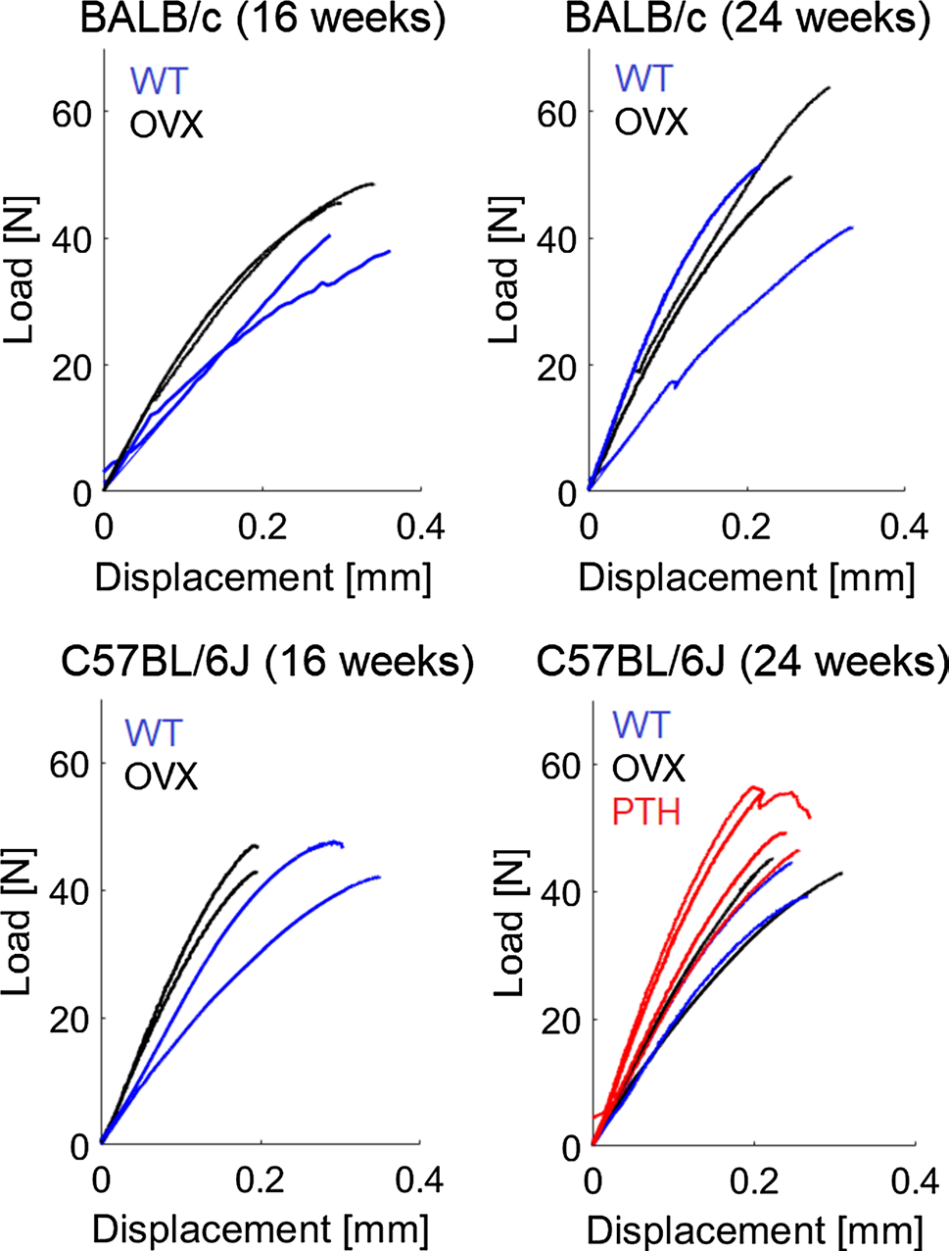
Load–displacement curves obtained for each specimen from 16-week (left) or 24-week (right) old female C57Bl/6 J (bottom) or Balb/C (top) mice. WT = wild type (blue), OVX = ovariectomized (black), PTH = treated with parathyroid hormone injections (red)

Bones were aligned with the axis of the loading machine (ElectroForce 3200, TA instruments) by using the reference blocks of embedding material in the custom-made fixation device (Fig. 1d). Ten preconditioning cycles were applied at 0.042 Hz between 1 and 4 N to achieve a steady viscoelastic state and to ensure stable boundary conditions during the test (Zhao et al. 2018). Afterwards, each bone was loaded in compression until failure at 0.03 mm/s (Holguin et al. 2013). Stiffness [N/mm] and failure load [N] were calculated as the slope of the linear portion and the maximum load from the load–displacement curve, respectively (Fig. 1d). Normalized stiffness and normalized failure load were calculated by dividing the stiffness and the failure load by the total BMC. The load–displacement curves obtained for the 20 specimens are reported in Fig. 2. In the figure, the initial toe region of the curves was removed by translating each curve of the amount calculated as the intersection between the initial linear part of the curve and the X-axis.

### Micro-Finite Element models

In order to replicate the experimental alignment in the microFE models, each image was rigidly rotated so that the longitudinal axis corresponded to the loading direction of the testing machine. The lower surface of the embedding material was identified from the microCT image in Amira (Amira 6.0.0, Thermo Fisher Scientific), exported into MATLAB and fitted to a plane (affine_fit function, https://www.mathworks.com/matlabcentral/fileexchange/43305-plane-fit, MATLAB Central File Exchange). Subsequently, the rotation angles to align the fitted plane to the horizontal direction were calculated and applied to the 3D image using Amira. After alignment, images were resampled using Lanczos interpolator (Birkhold et al. 2014). A Gaussian filter (kernel 3 × 3 × 3, standard deviation 0.65) was applied to reduce the high frequency noise (Bouxsein et al. 2010).

The image was segmented by using a single-level threshold, calculated as the average of the grey levels corresponding to the bone and background peaks in the image histogram (Christiansen 2016; Oliviero et al. 2017). A connectivity filter was applied in order to remove unconnected voxels (connectivity rule = 6, bwlabeln function, MATLAB).

Hexahedral meshes were obtained by using a custom-made MATLAB script (Chen et al. 2014, 2017) that converts each bone voxel into an eight-noded hexahedral element (Patel et al. 2014; Varga et al. 2020), resulting in 8–9 M elements per model. Tetrahedral meshes were obtained in Simpleware ScanIP (Synopsys, Mountain View, California, USA), by meshing the bone volume with ten-noded tetrahedral elements with maximum size of approximately 50 µm (coarseness factor equal to -50). A convergence study was performed on one model to identify the best mesh refinement (Supplementary material 2). The generated tetrahedral meshes included 2–2.5 M elements.

The finite elements of the models with homogeneous material properties (hexahedral or tetrahedral) were assigned a Young’s modulus equal to 14.8 GPa and a Poisson’s ratio of 0.3 (Oliviero et al. 2021). This value of Young’s modulus is in line with the mean elastic modulus measured from nanoindentation tests on the tibia of C57BL/6J and BALB/c female mice in a similar age range (Pepe et al. 2020). It also led to better FE predictions with respect to experimental data, compared to other common values used in the literature (Supplementary material 3). Heterogeneous material properties were assigned using a linear law to convert TMD into Young’s Modulus, adapted from the literature (Harrison et al. 2008). It was assumed that the minimum (500 mgHA/cc) and the maximum (1800 mgHA/cc) TMD were associated with the minimum and maximum elastic moduli (9 GPa and 23 GPa) measured by nanoindentation tests (Pepe et al. 2020), which resulted in the following equation:

$$E\left[\mathrm{MPa}\right]=\mathrm{TMD}[\mathrm{mgHA}/\mathrm{cc}]*10.7692+ 3.6154*{10}^{3}$$

This law was selected after testing four different laws in a subgroup of specimens (*N* = 8) and showed the best agreement with the experimental data (Supplementary material 4). The TMD range was divided into 450 intervals (MATLAB for hexahedral models, Simpleware ScanIP for tetrahedral models), resulting in approximately 450 materials per tibia, depending on the TMD distribution of each bone. The obtained distribution of elastic moduli had a peak at approximately 15 GPa, which was consistent with the modulus used for the homogeneous models. Lastly, hexahedral and tetrahedral models with specimen-specific homogeneous material properties were generated. Specimen-specific Young’s moduli were obtained from the average TMD of each specimen by using the linear law described above and were in the range of 12.7–15.4 GPa.

Uniaxial compression was simulated by fully constraining the distal end of the tibia and applying a displacement of 0.1 mm on each node of the proximal surface along the longitudinal direction. The apparent stiffness was calculated as the sum of reaction forces at the distal surface, divided by the applied displacement. For the estimation of failure load, the failure criterion proposed by Oliviero et al. (2021) was used: it was assumed that the tibia fails when 10% of the nodes reach a critical third principal strain of − 14,420 µε.

Models were solved on the high performance computing of the University of Sheffield (ShARC) using 16 cores and 32 GB/core of memory.

### Statistical analysis

Linear regression analysis was used to compare the experimental and predicted structural properties, and the predictions of the different models among each other. For each parameter and each model, the following regression parameters are reported: slope and intercept of the regression line, coefficient of determination (*R*^2^), root-mean-square error (RMSE), and percentage error (mean and standard deviation). For each regression, the two-tailed Student's t distribution (T.DIST.2T function, Excel) was used to determine whether slope and intercept of the regression line were significantly different from 1 and 0, respectively. Statistical significance was defined at *p* = 0.05.

## Results

The predictions of homogeneous microFE models with hexahedral mesh were already reported in Oliviero et al. (2021) and are reported here for comparison with the other models.

MicroFE models with hexahedral mesh and homogeneous material properties took approximately 20 min to solve, while those with heterogeneous material properties took approximately 90 min. Models with tetrahedral mesh and homogeneous material properties took approximately 10 min to solve, while those with heterogeneous material properties took approximately 20 min. An overview of the pre-processing, computation and post-processing times for the different models is reported in Table 2.

**Table 2:** Processing time for each step to generate and solve the different FE models

	Image pre-processing (min)	Meshing (min)	Simulation (min)	Post-processing (min)	Total (min)
Hexa-homo Hexa-homoTMD	10	15	20	5	50
Tetra-homo Tetra-homoTMD	15	15	10	5	45
Hexa-hete	10	30	90	5	135
Tetra-hete	15	40	20	5	80

*Hexa* hexahedral mesh, *Tetra* tetrahedral mesh, *Homo* homogeneous material properties, *Hete* heterogeneous material properties based on TMD

**Fig. 3:**
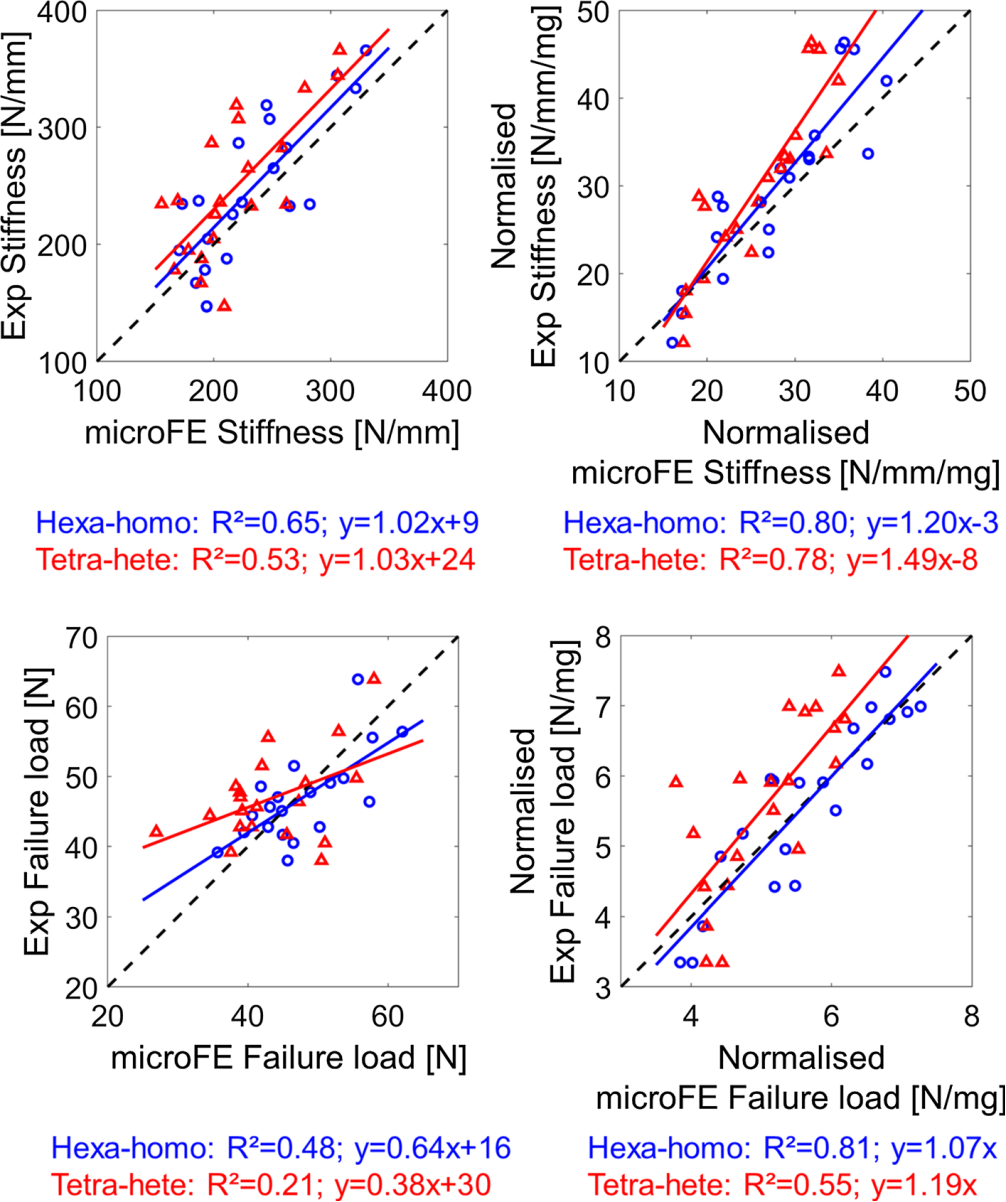
Regression analysis between the microFE predictions and experimental measurements of stiffness and failure load obtained with the simplest (hexa-homo) and most complex (tetra-hete) models. Normalized mechanical properties were obtained by dividing by the total bone mineral content (BMC, [mg]). Hexa = hexahedral mesh; Tetra = tetrahedral mesh; Homo = homogeneous material properties (E = 14.8GPa); Hete = heterogeneous material properties based on tissue mineral density

Regression analyses between the experimental measurements and microFE predictions of structural mechanical properties from the different models are reported in Table 3. In Fig. 3, regression analyses are reported for the simplest (Hexa-homo) and most complex (Tetra-hete) models with respect to experimental data. MicroFE predictions of stiffness were moderately correlated with experiments for all models: Hexa-homo models (*R*^2^ = 0.65, %Err = 14% ± 8%), Hexa-homoTMD (*R*^2^ = 0.56, %Err = 16% ± 11%), Tetra-homo (*R*^2^ = 0.62, %Err = 13% ± 11%), Tetra-homoTMD (*R*^2^ = 0.54, %Err = 15% ± 12%), Hexa-hete (*R*^2^ = 0.49, %Err = 17% ± 12%) and Tetra-hete (*R*^2^ = 0.53, %Err = 16% ± 12%). Normalized stiffness was strongly correlated with experimental measurements for all models (*R*^2^ = 0.75–0.80, Table 3).

**Table 3:** Regression parameters between experimental and predicted mechanical properties

	Slope	Intercept	*R*^2^	RMSE	*P* value	Error
Hexa-homo (*E* = 14.8GPa)						
S (N/mm)	1.02*	9**	0.65	38	< 0.001	14% ± 8%
S_norm (N/mm/mg)	1.20*	− 3**	0.80	4.58	< 0.001	14% ± 8%
Fu (N)	0.64	16	0.48	4.69	0.001	9% ± 6%
Fu_norm (N/mg)	1.07*	0**	0.81	0.56	< 0.001	9% ± 6%
Hexa-homo (TMD)						
S (N/mm)	1.08*	15**	0.56	42	< 0.001	16% ± 11%
S_norm (N/mm/mg)	1.51	− 9**	0.80	4.58	< 0.001	16% ± 11%
Fu [N]	0.57	21	0.35	5.26	0.006	12% ± 6%
Fu_norm (N/mg)	1.46	− 2	0.79	0.58	< 0.001	12% ± 6%
Tetra-homo (E = 14.8GPa)						
S (N/mm)	0.95*	16**	0.62	39	< 0.001	13% ± 11%
S_norm (N/mm/mg)	1.11*	− 2**	0.78	4.81	< 0.001	13% ± 11%
Fu (N)	0.61	18	0.43	4.94	0.002	10% ± 7%
Fu_norm (N/mg)	1.09*	0**	0.77	0.61	< 0.001	10% ± 7%
Tetra-homo (TMD)						
S (N/mm)	1.01*	20**	0.54	43	< 0.001	15% ± 12%
S_norm (N/mm/mg)	1.40	− 8**	0.78	4.84	< 0.001	15% ± 12%
Fu (N)	0.55	21	0.33	5.34	0.008	11% ± 7%
Fu_norm (N/mg)	1.36	− 2**	0.79	0.59	< 0.001	11% ± 7%
Hexa-hete						
S (N/mm)	0.99*	37**	0.49	45	0.001	17% ± 12%
S_norm (N/mm/mg)	1.49	-8**	0.75	5.14	< 0.001	17% ± 12%
Fu (N)	0.55	23	0.32	5.37	0.009	12% ± 7%
Fu_norm (N/mg)	1.41	− 2**	0.76	0.63	< 0.001	12% ± 7%
Tetra-hete						
S (N/mm)	1.03*	24**	0.53	44	< 0.001	16% ± 12%
S_norm (N/mm/mg)	1.49	− 8**	0.78	4.77	< 0.001	16% ± 12%
Fu (N)	0.38	30	0.21	5.80	0.042	15% ± 10%
Fu_norm (N/mg)	1.19*	0**	0.55	0.86	< 0.001	15% ± 10%

*S* stiffness (N/mm), *S_norm* normalized stiffness (N/mm/mg), *Fu * failure load (N), *Fu_norm* normalized failure load (N/mg), *Hexa* hexahedral mesh, *Tetra* tetrahedral mesh, *Homo* homogeneous material properties (either *E* = 14.8 GPa or sample-specific E based on the average tissue mineral density, TMD), *Hete* heterogeneous material properties based on TMD

*Indicates that the slope was not significantly different from 1 (*p* value > 0.05). **Indicates that the intercept was not significantly different from 0 (*p* value > 0.05)

The highest correlation between experimental and predicted failure load was found for Hexa-homo models (*R*^2^ = 0.48, %Err = 9% ± 6%). Correlations were lower for Hexa-homoTMD models (*R*^2^ = 0.35, %Err = 12% ± 6%), Tetra-homo (*R*^2^ = 0.43, %Err = 10% ± 7%), Tetra-homoTMD (*R*^2^ = 0.33, %Err = 11% ± 7%), Hexa-hete (*R*^2^ = 0.32, %Err = 12% ± 7%) and Tetra-hete (R^2^ = 0.21, %Err = 15% ± 10%) models. Normalized failure load was strongly correlated with experimental measurements for five of the models (*R*^2^ = 0.76–0.81, Table 3), while the lowest correlation was found for Tetra-hete models (*R*^2^ = 0.55, Table 3).

**Fig. 4:**
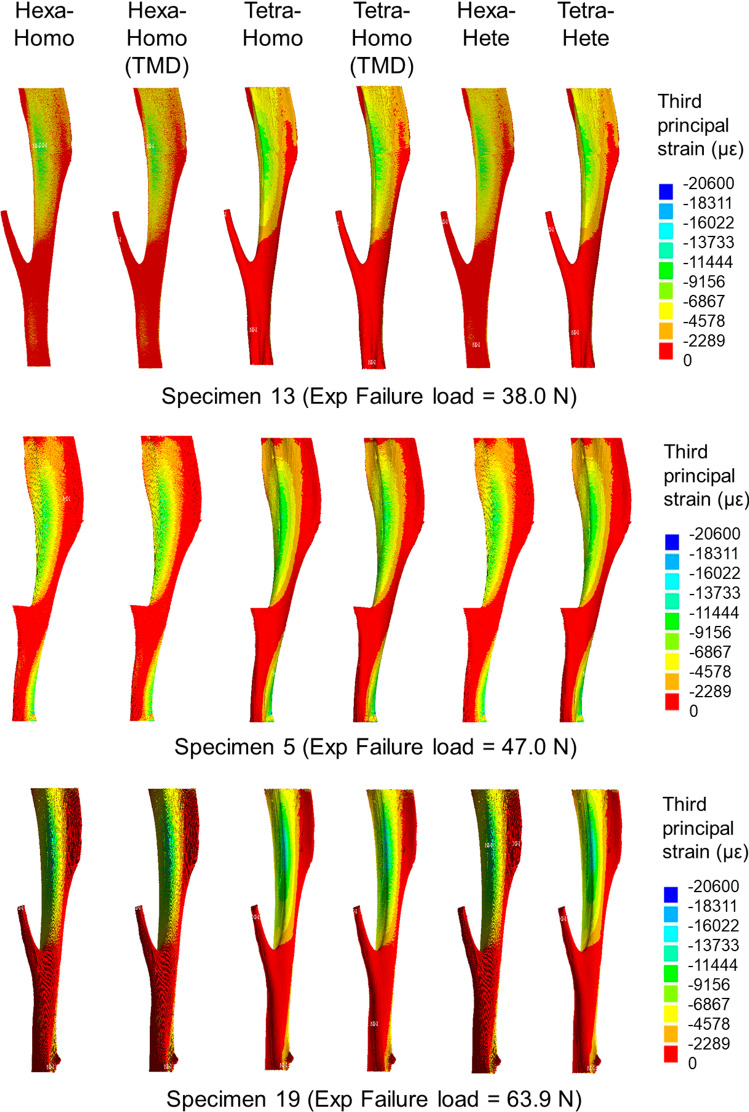
Strain distributions for three specimens, for which the highest, lowest and average failure load was measured. Hexa = hexahedral mesh; Tetra = tetrahedral mesh; Homo = homogeneous material properties (either E = 14.8GPa or sample-specific E based on the average tissue mineral density, TMD); Hete = heterogeneous material properties based on TMD

**Fig. 5:**
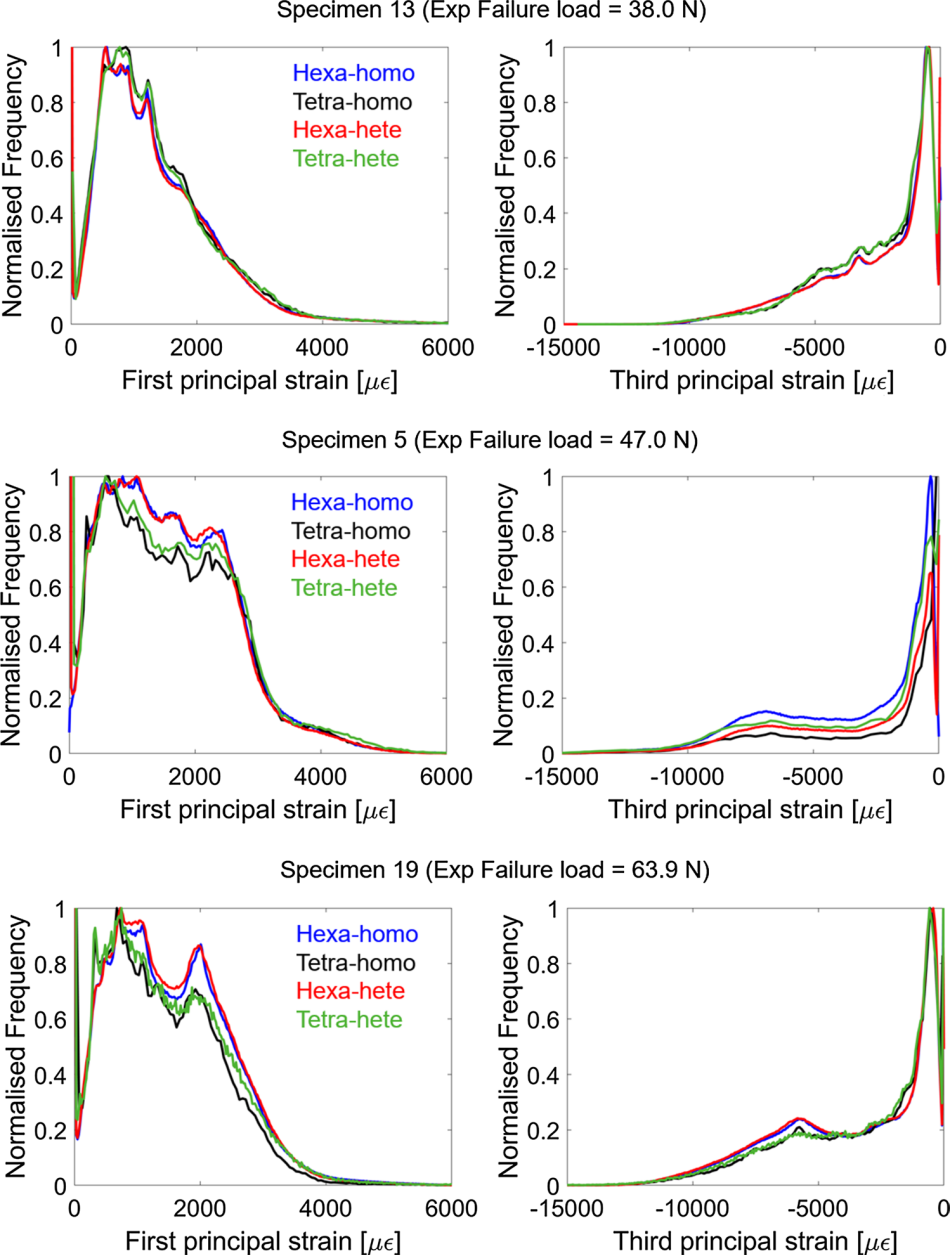
Frequency plots of first and third principal strains for three specimens, for which the highest, lowest and average failure load was measured, respectively. Hexa = hexahedral mesh; Tetra = tetrahedral mesh; Homo = homogeneous material properties (E = 14.8GPa; models with specimen-specific E were not reported for clarity); Hete = heterogeneous material properties based on tissue mineral density

Spatial distributions of strains and strain histograms for three specimens (lowest, highest and average measured failure load) are reported in Figs. 4 and 5. Spatial distributions of strains (Fig. 4) and strain histograms (frequency plots in Fig. 5) were similar among models, with peaks of strains located at the postero-lateral apex and on the antero-medial surface towards the distal end of the tibia (Fig. 4).

**Fig. 6:**
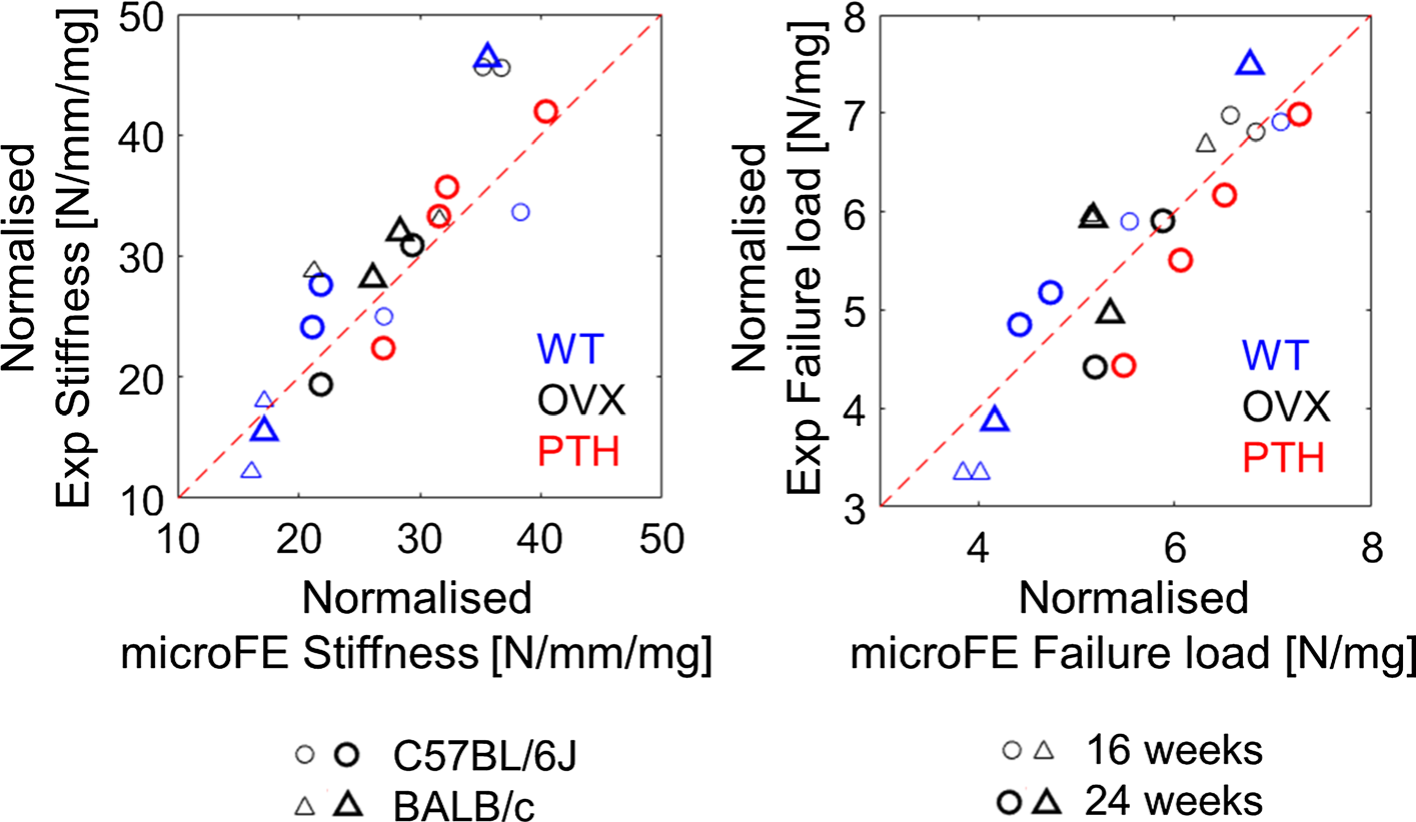
Experimental and predicted (hexahedral mesh, homogeneous material properties, E = 14.8 GPa) structural properties for the different groups. WT = wild type, OVX = ovariectomized, PTH = treated with parathyroid hormone injections; circle = C57BL/6J, triangle = BALB/c; small marker = 16 weeks of age, large marker = 24 weeks of age

No particular trend was observed for the predictions if the data were split for the different mouse strains, intervention groups or age (Fig. 6).

**Fig. 7:**
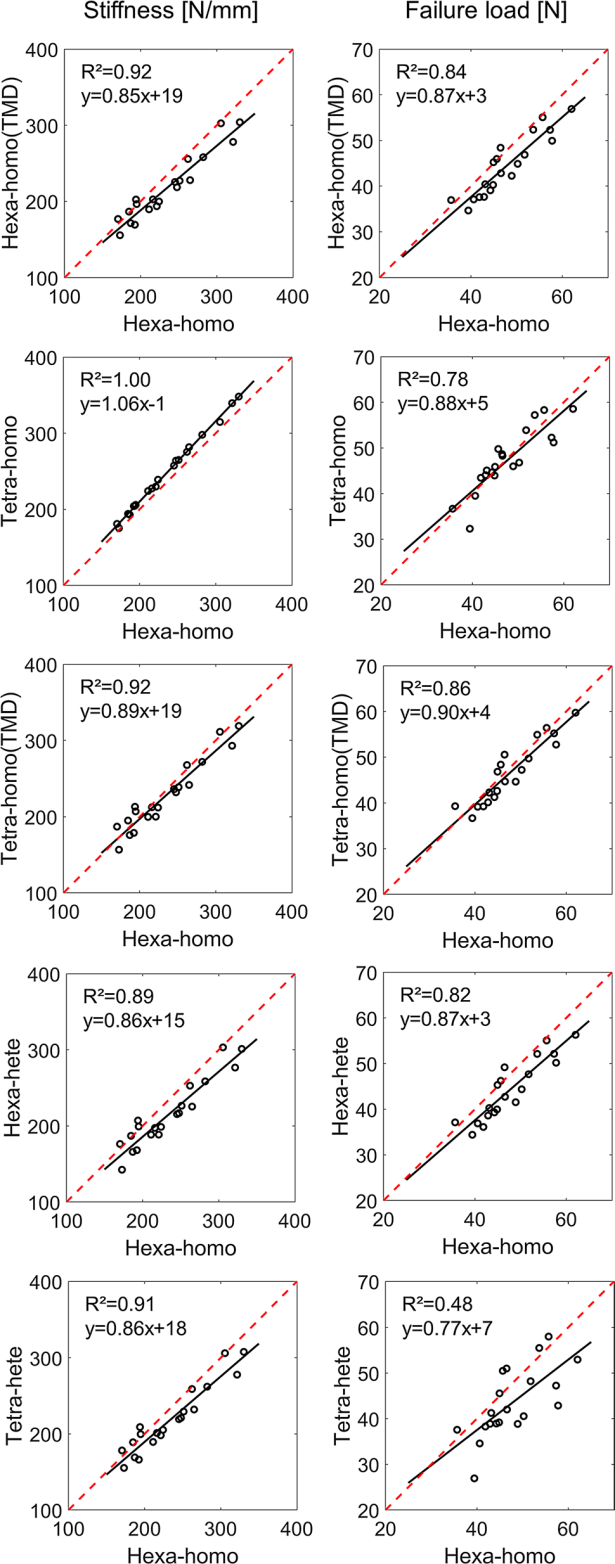
Regression analyses for stiffness and failure load estimated by the different models with respect to the Hexa-homo one. Hexa = hexahedral mesh; Tetra = tetrahedral mesh; Homo = homogeneous material properties (either E = 14.8 GPa or specimen-specific E based on the average tissue mineral density, TMD); Hete = heterogeneous material properties based on tissue mineral density

Regression analyses between the different model types are reported in Fig. 7. The predicted apparent stiffness and failure load from the Hexa-homo models were in most cases strongly correlated with those obtained from the other models (apparent stiffness: *R*^2^ = 0.89–1.00; failure load: *R*^2^ = 0.78–0.86 for four models, *R*^2^ = 0.48 for Hexa-homo versus Tetra-hete, Fig. 7).

## Discussion

In this study, the abilities of different modelling approaches to predict the structural mechanical properties of the mouse tibia have been evaluated against experimental measurements.

The experimental measurements of compressive failure load were higher than those reported in the literature. To the authors’ knowledge, only one study measured the failure load of the mouse tibia in compression for age range, sex and strain similar to the one considered in this study (Holguin et al. 2013). The differences in the range (38–64 N in our study vs. 18–32 N in that study) could be due to differences in the bone alignment, the fixation of the bone in the testing machine and the loading procedure.

The six model types, based on in vivo microCT images, showed similar accuracy (Fig. 3, Supplementary material 5) and consistent strain predictions (Figs. 4, 5). From the analyses of the frequency plots of first and third principal strains, it seems that the local strain distributions were mainly affected by the mesh type rather than material properties (Fig. 5). This is in line with the expected concentrations of stresses and strains in the hexahedral mesh compared to the smoother tetrahedral mesh. Nevertheless, excellent correlation was found between the structural properties predicted with hexahedral or tetrahedral models, highlighting that the geometry was consistently modelled with the two mesh types. However, such differences in local strains may affect the prediction of bone remodelling based on local mechanoregulation (Cheong et al. 2020a, b) and should be considered when designing multi-scale computational models. The material properties assignment mainly affected the correlation between experimental and predicted structural properties, as models with the same material properties resulted in similar slopes of the regression lines (Table 3, Supplementary material 5). Decreasing the modulus had the effect of increasing the slope of the regression line (Supplementary material 3). The predictive accuracy decreased with the model complexity (average error increased by 3–5% by including the material heterogeneity). This is probably due to the uncertainties in the evaluation of the local TMD values from in vivo microCT scans, which are affected by image artefacts, such as beam hardening, that propagate in the FE model in function of the assignment of the heterogeneous material properties through the chosen constitutive law. It is interesting to note that a similar consideration in a quite different study was concluded by Kluess et al. (2019) when analysing the uncertainties in FE modelling approaches for the human femur by different laboratories. The model using the simplest definition for material properties (homogeneous) was among those that achieved more accurate results compared to more complex models. The highest correlations between microFE predictions and experimental measurements were found for the hexahedral models with homogeneous material properties (*R*^2^ = 0.65 for stiffness, *R*^2^ = 0.48 for failure load). The correlations with experimental measurements improved when considering the size and overall mineralization of each specimen (*R*^2^ = 0.80 for stiffness normalized by the total bone mineral content, *R*^2^ = 0.81 for normalized failure load). The lowest average errors in the stiffness prediction were found for tetrahedral models with homogeneous material properties (13% ± 11%), although they were very similar to those obtained for Hexa-homo models (14% ± 8%). The lowest average errors in the failure load prediction were found for Hexa-homo models (9% ± 6%). These results suggest that the geometry and loading conditions are the main factors driving the structural properties of the mouse tibia in compression. This is in line with the lack of correlation between structural properties and total bone mineral content or local TMD in subregions of the bone (Oliviero et al. 2021). A similar result has been reported in a previous study on the mouse femur (Varga et al. 2020), where it was suggested that the mechanical properties of a bone structure mainly made of cortical tissue are driven by its geometry rather than the local tissue mineralization, if in reasonable range. The lower accuracy associated with the heterogeneous models is likely due to the assumptions applied for converting TMD into Young’s modulus. Different relationships are available in the literature to estimate Young’s modulus from TMD, based on bone specimens from different species (Austman et al. 2009; Currey 1988; Easley et al. 2010; Harrison et al. 2008). In this study, after a preliminary analysis where different approaches were compared (Supplementary material 4), one of these methods was selected and adapted to the mouse tibia using nanoindentation data. Although the average modulus obtained was consistent with previously reported data (Birkhold et al. 2016; Yang et al. 2014), the accuracy of the estimated local Young’s modulus is unclear, which may be the reason for the larger data scatter obtained for heterogeneous models.

The correlations found in this study between experimental and predicted mechanical properties were generally lower compared to previous validation studies on different mouse bone structures (*R*^2^ = 0.62–0.89 for the mouse vertebra in compression (Nyman et al. 2015), *R*^2^ = 0.93 for the mouse femur in four-point bending (Varga et al. 2020)). This difference can be due to the complex stress and strain distributions within the different regions of the bone, resulting from the combination of local compression and bending and due to the geometrical properties of the tibia (e.g. large aspect ratio and natural curvature). In order to replicate the experimental loading conditions, the microCT images were acquired after embedding the tibia extremities in resin, in order to use the embedding material as reference surface for matching the loading direction in the models and in the experiments. Nevertheless, the limitation of this approach is that any compliance in the embedding material was not considered, as it was assumed that the displacement applied to the top surface of the embedding material is perfectly transmitted to the upper surface of the tibia free length. This is an idealization that may affect the measured and estimated structural stiffness of the mouse tibia. Assuming that the metal components of the machine did not affect drastically the compliance of the setup, the embedding material is estimated to have a structural stiffness of approximately 10,200 N/mm, which would contribute to the bone stiffness of approximately 4.9%. Nevertheless, we have not compensated for this value in the predictions as the problem is more complex, with portions of bone, growth plate and embedding material in the regions adjacent to the modelled bone. Another limitation of this study is that the tested tibiae covered a relatively small range of properties. In order to increase the range in mechanical properties, tibiae from different groups of mice were included in the study. Nevertheless, a larger age range may have helped in understanding the potential of the approach for studies including younger or older mice. Lastly, even though in this study material heterogeneity and smooth models were considered, the models could be improved in the future by accounting for material nonlinearities (Stipsitz et al. 2020) and poroelastic behaviour of the bone (Pereira et al. 2015). These properties of the bone material could possibly have a larger impact on the structural mechanical behaviour compared to the local heterogeneity.

In summary, in this study different microFE modelling approaches have been evaluated for the non-invasive prediction of the mouse tibia structural mechanical properties from in vivo microCT images. Hexahedral models with homogeneous material properties provided the best compromise between prediction accuracy and time, given that they require minimal operator-dependent procedures, and therefore can be generated and solved almost automatically. Nevertheless, further studies testing different constitutive laws and assignment of material properties from the TMD distributions should be performed in order to generalize this result.

## Supplementary Information

Below is the link to the electronic supplementary material.

Supplementary material 1 (DOCX 1100 KB)

## Data Availability

Upon acceptance of the manuscript, a link to Figshare will be added with instructions about how to access the data used in this study (https://bit.ly/3aasDVw).
