# Supplementary material for: Non-invasive prediction of the mouse tibia mechanical properties from microCT images: comparison between different finite element models
Source: Biomech Model Mechanobiol. 2021 Feb 1;20(3):941–55. doi: 10.1007/s10237-021-01422-y (PMC8154847; doi:10.1007/s10237-021-01422-y)
Supplement: Supplementary file 1 — Supplementary material 1 (DOCX 1100 KB) [file 10237_2021_1422_MOESM1_ESM.docx]

**Supplementary material**

**Non-invasive prediction of the mouse tibia mechanical properties from microCT images: comparison between different Finite Element models**

S. Oliviero ^a,b^, M. Roberts ^c^, R. Owen ^b,d,e^, G. C. Reilly ^b,d^, I. Bellantuono ^a,b,f^, E. Dall’Ara ^a,b, f^*

^a^ Department of Oncology and Metabolism, Mellanby Centre for bone Research, University of Sheffield, UK

^b^ INSIGNEO Institute for *in silico* Medicine, University of Sheffield, UK

^c^ Department of Mechanical Engineering, University of Sheffield, UK

^d^ Department of Materials Science and Engineering, University of Sheffield, UK

^e^ Regenerative Medicine and Cellular Therapies, School of Pharmacy, University of Nottingham Biodiscovery Institute, University Park, UK

^f^ Healthy Lifespan Institute, Department of Oncology and Metabolism, The Medical School, University of Sheffield, UK

****Corresponding Author.***

Name Surname: Enrico Dall’Ara

Telephone number: +44 (0) 114 2226175

E-mail: e.dallara@sheffield.ac.uk

**Supplementary material 1**

**A1. Properties of the specimens**

Properties of the mouse tibiae used in this study have been presented in a previous study (Oliviero et al., 2020) and are reported in Table A1.

| N | Strain | Group | Age  [weeks] | Side | BMC  [mg] | TMD  [mgHA/cc] | BV/TV  [-] | Experimental  Stiffness  [N/mm] | Experimental Failure  Load  [N] |
| --- | --- | --- | --- | --- | --- | --- | --- | --- | --- |
| 1 | C57BL/6J | WT | 16 | Right | 7.12 | 871 | 0.62 | 178 | 42.0 |
| 2 | C57BL/6J | WT | 16 | Right | 6.91 | 846 | 0.58 | 233 | 47.8 |
| 3 | C57BL/6J | WT | 24 | Left | 8.58 | 918 | 0.60 | 237 | 44.4 |
| 4 | C57BL/6J | WT | 24 | Left | 8.07 | 1087 | 0.59 | 195 | 39.2 |
| 5 | C57BL/6J | OVX | 16 | Right | 6.74 | 873 | 0.58 | 307 | 47.0 |
| 6 | C57BL/6J | OVX | 16 | Left | 6.28 | 866 | 0.57 | 287 | 42.8 |
| 7 | C57BL/6J | OVX | 24 | Left | 9.68 | 890 | 0.56 | 188 | 42.8 |
| 8 | C57BL/6J | OVX | 24 | Left | 7.63 | 893 | 0.55 | 236 | 45.1 |
| 9 | C57BL/6J | PTH | 24 | Left | 10.46 | 918 | 0.64 | 234 | 46.4 |
| 10 | C57BL/6J | PTH | 24 | Left | 10.24 | 923 | 0.62 | 366 | 56.4 |
| 11 | C57BL/6J | PTH | 24 | Left | 7.95 | 906 | 0.61 | 265 | 49.1 |
| 12 | C57BL/6J | PTH | 24 | Left | 7.95 | 849 | 0.61 | 333 | 55.5 |
| 13 | BALB/c | WT | 16 | Right | 11.36 | 1047 | 0.66 | 205 | 38.0 |
| 14 | BALB/c | WT | 16 | Right | 12.10 | 1094 | 0.65 | 147 | 40.5 |
| 15 | BALB/c | WT | 24 | Right | 10.79 | 1046 | 0.70 | 167 | 41.7 |
| 16 | BALB/c | WT | 24 | Right | 6.88 | 925 | 0.69 | 319 | 51.5 |
| 17 | BALB/c | OVX | 16 | Right | 8.15 | 897 | 0.65 | 235 | 48.6 |
| 18 | BALB/c | OVX | 16 | Left | 6.83 | 949 | 0.70 | 226 | 45.6 |
| 19 | BALB/c | OVX | 24 | Right | 10.77 | 1023 | 0.73 | 344 | 63.9 |
| 20 | BALB/c | OVX | 24 | Right | 10.04 | 1003 | 0.68 | 282 | 49.7 |

***Table A1****. Properties of the tested mouse tibiae. WT = wild type, OVX = ovariectomised, PTH = treated with parathyroid hormone injections. Parameters reported: BMC = bone mineral content, TMD = tissue mineral density, BV/TV = bone volume fraction.*

**Supplementary material 2**

**A2. Convergence study**

A microCT image of a mouse tibia (C57BL/6J, wild type, 22 weeks of age) was segmented and meshed with quadratic tetrahedral elements. Seven models were generated with increasing mesh refinement from maximum element size of 25 µm to 80 µm (Table A2). The model with element size of 25 µm was used as reference. Uniaxial compression was simulated by fully constraining the distal end of the tibia and applying a 0.1 mm displacement at the proximal end in the longitudinal direction. Each model was compared to the reference one by calculating the difference between the model predictions for the following parameters: apparent stiffness, failure load, displacement along z direction and third principal strain at the location of maximum third principal strain (Table A2).

| Max element size (µm) | Number of elements | Difference  with respect to reference model  (%) | | | |
| --- | --- | --- | --- | --- | --- |
|  |  | Stiffness | Failure load | Displacement along *z* | Third principal strain |
| 25 | 7193765 | Reference | Reference | Reference | Reference |
| 30 | 4161922 | -1.94 | -2.16 | -0.01 | -4.41 |
| 40 | 1752865 | 1.49 | 1.11 | -0.04 | -4.32 |
| 50 | 897820 | 1.75 | 1.20 | -0.07 | -3.68 |
| 60 | 518613 | 9.51 | 9.08 | -0.16 | -6.85 |
| 70 | 326458 | -4.55 | -5.36 | -0.17 | -8.09 |
| 80 | 218574 | -1.00 | -1.30 | -0.22 | -13.56 |

***Table A2.*** *Results of convergence study.*

Convergence was obtained for maximum element size of 50 µm. This result was consistent with the data reported in the literature, where it has been shown that tetrahedral models of mouse tibia are at convergence with 1500000-1800000 elements (Razi et al., 2015, Yang et al., 2014).

**Supplementary material 3**

**A3. Effect of homogeneous elastic modulus assignment on stiffness**

Regression analyses with respect to experimental data are reported for hexahedral models with three different values of homogeneous elastic modulus (Fig A3, Table A3). Three different values of elastic modulus were considered: 14.8 GPa (Oliviero et al., 2020), specimen-specific E based on the average tissue mineral density (TMD), and 17 GPa, which has been used to model the tibia in other studies (Pereira et al., 2015, Razi et al., 2015). The best correlation with experimentally measured stiffness and the lowest errors were found for E = 14.8 GPa.


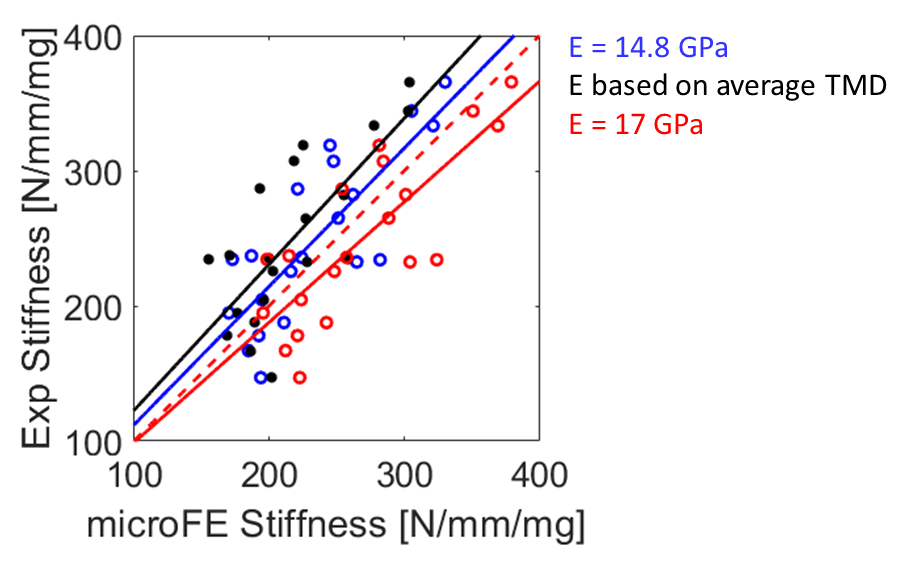


***Figure A3****. Regression analysis between the microFE predictions and experimental measurements of stiffness for hexahedral models with homogeneous materials properties. Three different values of elastic modulus are reported: 14.8 GPa (Oliviero et al., 2020), specimen-specific E based on the average TMD, and 17 GPa, which has been used to model the tibia in other studies (Pereira et al., 2015, Razi et al., 2015).*

| Regression  analysis with experimental stiffness | E = 14.8 GPa | E based on average TMD | E = 17 GPa |
| --- | --- | --- | --- |
| R^2^ | 0.65 | 0.56 | 0.65 |
| Slope | 1.02 | 1.08 | 0.89 |
| Intercept | 9 | 15 | 9 |
| Error | 14% ± 8% | 16% ± 11% | 16% ± 13% |

***Table A3.*** *Regression analyses for hexahedral models with homogeneous material properties. Three different values of elastic modulus are reported: 14.8 GPa, specimen-specific E based on the average TMD and 17 GPa.*

**Supplementary material 4**

**A4. Effect of heterogeneous elastic modulus assignment on stiffness**

For the assignment of heterogeneous material properties, four different laws to convert local tissue mineral density (TMD) into elastic modulus (E) were compared:

E calculated based on the estimated calcium concentration (Currey, 1988, Gross et al., 2012):

$\log E[GPa]= -5.22+2.71\log(Ca);Ca[mg/g]=0.2 TMD[mgHA/cc]$

E estimated based on the power law proposed by (Easley et al., 2010), which was applied in (Yang et al., 2014) for FE models of the mouse tibia:

$$E[MPa]=0.1127 {TMD[mgHA/cc]}^{1.746}$$

E estimated by assuming a linear law based on nanoindentation data (adapted from (Harrison et al., 2008)). It was assumed that the minimum (500 mgHA/cc) and the maximum (1800 mgHA/cc) TMD were associated with the minimum and maximum elastic moduli (9 GPa and 23 GPa) measured by nanoindentation (Pepe et al., 2020):

$$E[MPa]=TMD[mgHA/cc]*10.7692+ 3.6154*{10}^{3}$$

E estimated based on the power law proposed by (Austman et al., 2009), which was adapted by (Razi et al., 2015) for FE models of the mouse tibia:

$$E= E_{max} \frac{{TMD}^{1.5}}{{TMD}_{max}^{1.5}}; E_{max}= 17GPa; {TMD}_{max}=1141mgHA/cc$$

The estimated E for each law is reported in Fig A4.

Stiffness was estimated for a subgroup of samples (N=8) using each of the different laws. Regression parameters and errors obtained by comparing the microFE stiffness with experimental measurements are reported in Table A4. The highest correlation and lowest errors were obtained for the linear law.


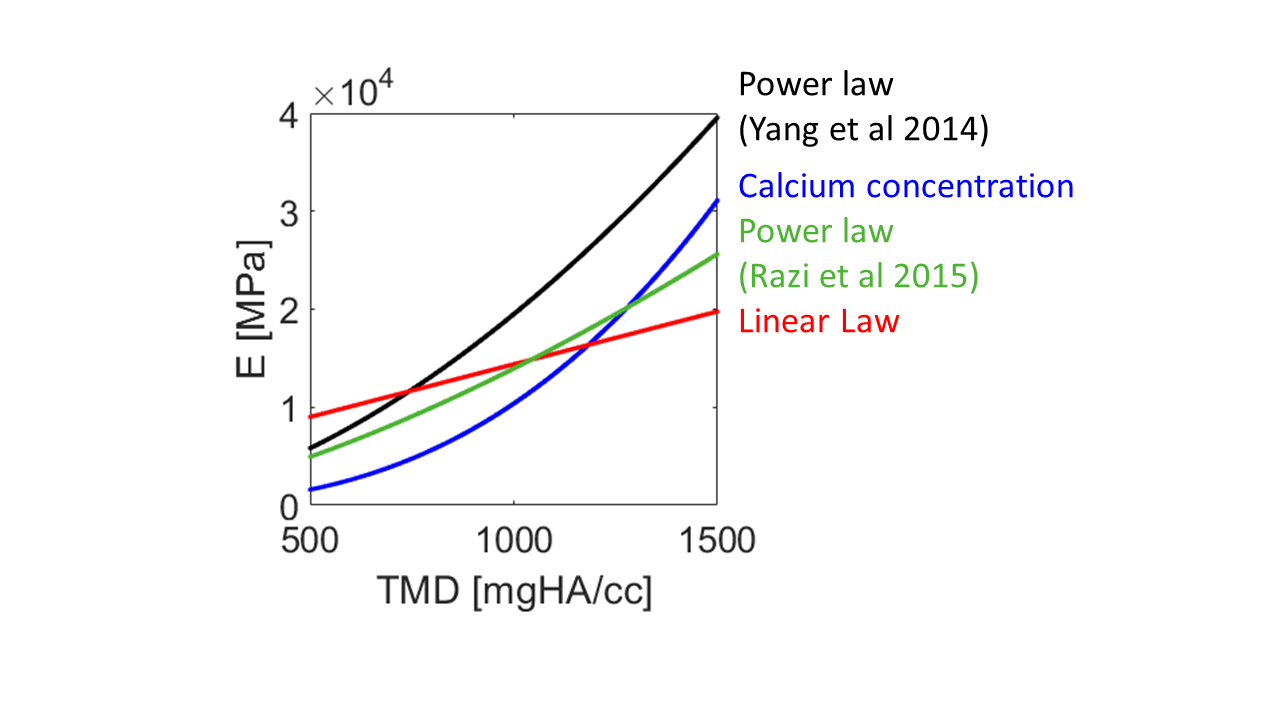


***Figure A4****. Elastic modulus obtained from local tissue mineral density based on different laws.*

| Regression  analysis with experimental stiffness | Calcium concentration | Power law  (Yang et al., 2014) | Linear law | Power law  (Razi et al., 2015) |
| --- | --- | --- | --- | --- |
| R^2^ | 0.32 | 0.59 | 0.83 | 0.66 |
| Slope | 0.97 | 0.81 | 1.34 | 1.22 |
| Intercept | 80 | -4 | -61 | -23 |
| Error | 36% ± 11% | 30% ± 31% | 12% ± 9% | 17% ± 12% |

***Table A4.*** *Regression analyses between experimental stiffness and FE predictions using hexahedral models with heterogeneous material properties, based on different laws to convert TMD into E.*

**Supplementary material 5**

**A5. Regression plots for the different models**

Regression analyses with respect to experimental data are reported for the different models in Fig A5.


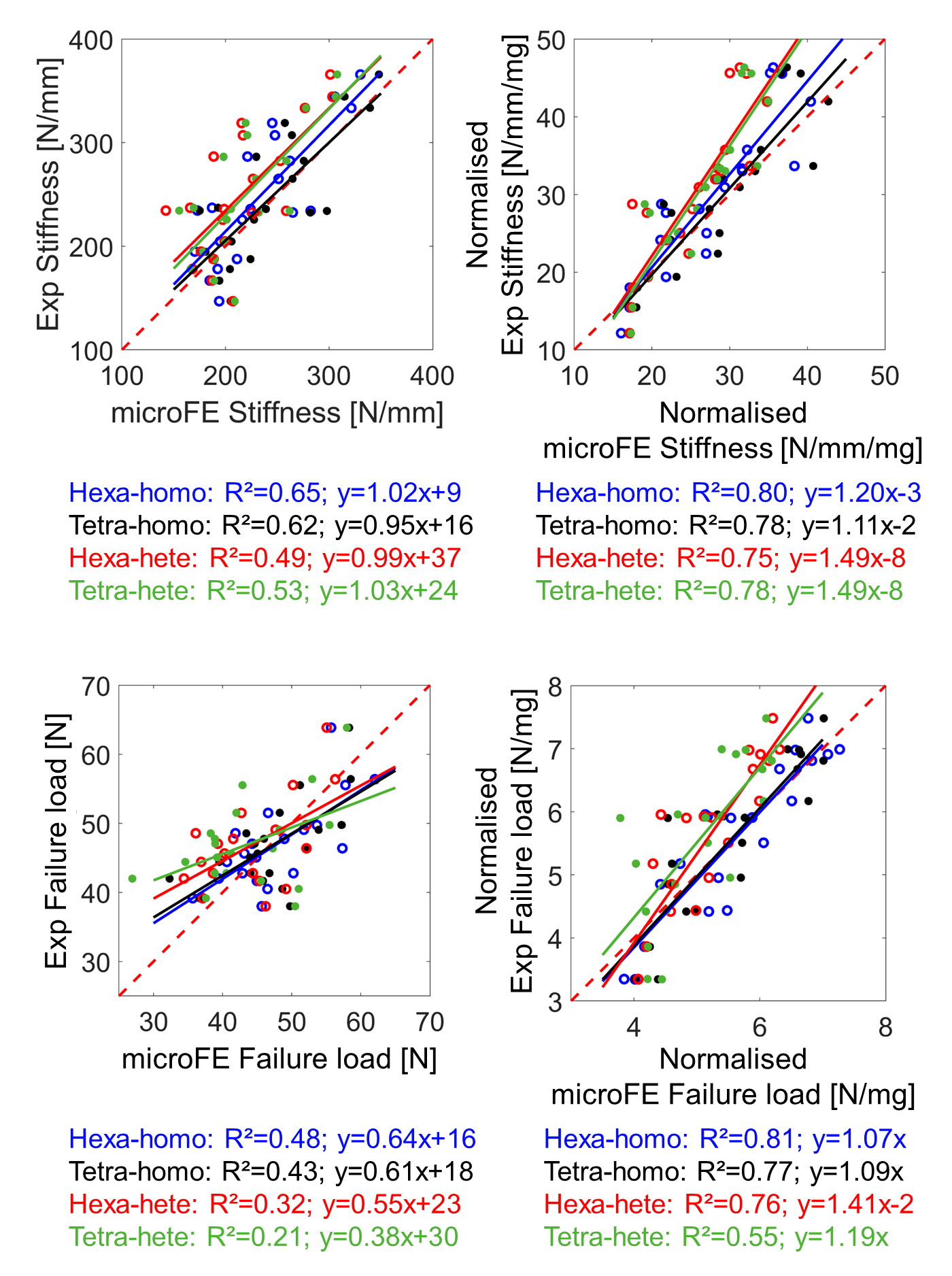


***Figure A5****. Regression analysis between the microFE predictions and experimental measurements of stiffness and failure load obtained with the different models. Normalized mechanical properties were obtained by dividing by the total bone mineral content (BMC, [mg]). Hexa = hexahedral mesh; Tetra = tetrahedral mesh; Homo = homogeneous material properties (E=14.8GPa, models with specimen-specific E were not reported for clarity); Hete = heterogeneous material properties based on tissue mineral density.*
